# Population-specific climate sensitive top height curves and their applications to assisted migration

**DOI:** 10.1007/s10342-024-01694-w

**Published:** 2024-05-13

**Authors:** Dawei Luo, Gregory A. O’Neill, Yuqing Yang, Esteban Galeano, Tongli Wang, Barb R. Thomas

**Affiliations:** 1 Department of Renewable Resources, University of Alberta, 442 Earth Science Buildings, Edmonton, AB T6G 2E3 Canada; 2 Department of Forest and Conservation Sciences, University of British Columbia, Vancouver, BC V6T 1Z4 Canada; 3 Kalamalka Forestry Centre, BC Ministry of Forests, 3401 Reservoir Road, Vernon, BC V1B 2C7 Canada; 4 Lands Planning Branch, Alberta Environment and Parks, 3 Floor, Petroleum Plaza South Tower, 9915 - 108 Street, Edmonton, AB T5K 2G8 Canada; 5 Department of Forestry, Mississippi State University, Thompson Hall, Rm 351, Starkville, Mississippi 39762 USA

**Keywords:** Assisted migration, Climate change, Top height, Universal Response Function, White spruce, Lodgepole pine

## Abstract

Growth and yield (G&Y) of forest plantations can be significantly impacted by maladaptation resulting from climate change, and assisted migration has been proposed to mitigate these impacts by restoring populations to their historic climates. However, genecology models currently used for guiding assisted migration do not account for impacts of climate change on cumulative growth and assume that responses of forest population to climate do not change with age. Using provenance trial data for interior lodgepole pine (*Pinus contorta* subsp. *latifolia* Douglas) and white spruce (*Picea glauca* (Moench) Voss) in western Canada, we integrated Universal Response Functions, representing the relationship of population performance with their provenance and site climates, into top height curves in a G&Y model (Growth and Yield Projection System, GYPSY) to develop population-specific climate sensitive top height curves for both species. These new models can estimate the impact of climate change on top height of local populations and populations from a range of provenances to help guide assisted migration. Our findings reveal that climate change is expected to have varying effects on forest productivity across the landscape, with some areas projected to experience a slight increase in productivity by the 2050s, while the remainder are projected to face a significant decline in productivity for both species. Adoption of assisted migration, however, with the optimal populations selected was projected to maintain and even improve productivity at the provincial scale. The findings of this study provide a novel approach to incorporating assisted migration approaches into forest management to mitigate the negative impacts of climate change.

## Introduction

Forest populations normally display their highest fitness near their geographic or climatic origin. This phenomenon, known as local adaptation, has been extensively documented in widespread forest tree species (Leimu and Fischer 2008). Local adaptation is of great significance as it plays a fundamental role in our understanding of evolutionary, population, and conservation biology, and in formulating reforestation strategies intended to address climate change. Local adaptation also informs seed transfer practices in resource management and conservation of forest ecosystems to maintain population health, productivity, and resilience, especially in the face of rapidly changing environmental conditions (Ying and Yanchuk 2006).

The principle of “local is best” has long been upheld for seed transfer guidance in forest management. However, the escalating occurrence of maladaptation events in forest stands related to climate change has placed significant strain on geographically local populations (Hogg et al. 2017; Sperlich et al. 2020). Rapid climate change, relative to the long generation times and slow rate of migration and adaptation of temperate and boreal forests (Malcolm et al. 2002), significantly heightens the risk of maladaptation of naturally established forests and stands planted with local seed sources (Pedlar et al. 2012).

Local adaptation, which is typically assessed in provenance trials, is used to identify the need for assisted migration. Using data from provenance trials of interior lodgepole pine (*Pinus contorta* subsp. *latifolia* Douglas) across British Columbia and the southern Yukon, Canada, previous studies have investigated the feasibility of applying Universal Transfer Functions (UTFs) (O'Neill et al. 2008; Leites et al. 2012b) and a Universal Response Function (URF) (Wang et al. 2010) to predict growth responses of populations to a range of climate conditions, and therefore help provide guidance for assisted migration. In UTFs, population traits (e.g., height) are related to provenance climate and climate transfer distance (climate difference between provenance and site), while in URFs, these traits were related to provenance and site climate. In these UTF and URF models, test site climate is considered a reflection of the short-term environmental and genetic-by-environment interaction effect on the phenotype, while provenance climate is considered a reflection of the long-term genetic effect of natural selection on the phenotype (Leites et al. 2012b). Although developed using different approaches, URFs and UTFs perform similarly in understanding forest population responses to climate (Zhao and Wang 2023).

Although UTFs and URFs are mature tools, and have been used widely, they still have several limitations in predicting the impact of climate change or seed transfer on forest productivity. First, UTF and URF functions use data from a single age, requiring the assumption that population responses to climate do not vary with tree age. Second, these functions are usually developed from relatively young (e.g., 5–20-year-old) provenance tests; therefore, the growth of the tested populations represents the impact of climate change on the population growth rate at their test climate and not the impact of climate change on the cumulative growth of local populations in operational plantations when the plantations reach the test climate or rotation age (O'Neill and Nigh 2011). These limitations could be addressed by growth and yield (G&Y) models, in which cumulative growth over age is an essential component. For instance, Pooled Transfer Functions, which relate standardized growth pooled across multiple sites and populations to climate transfer distance, were merged with a height over age curve for lodgepole pine (*P. contorta* subsp. *latifolia* Douglas) and used in productivity prediction and optimal seed source selection (O'Neill and Nigh 2011; Nigh 2014).

Growth and yield models are a widely used tool for estimating forest productivity and their predictions play a key role in supporting sustainable forest management decisions. Top height, which is defined as the average height of the dominant trees in the stand at a given age, and site index (SI), which normally refers to top height at a reference age (i.e., age-50 or age-80), often play roles of stand productivity indicators (Skovsgaard and Vanclay 2008) and are main drivers for many G&Y models (Mitchell 1975; Huang et al. 2009). Consequently, height-over-age curves are a fundamental component in these G&Y models. Although effects of climate change on G&Y have been investigated by adjusting impacts on live crown (Yang et al. 2019), height increment, and diameter increment (Yang et al. 2019), the incorporation of climate effects into these G&Y models is primarily associated with naturally regenerated forests and plantations using local seed sources. In addition, it is also of great interest to explore the application of G&Y models in forest management strategies when considering climate change risk, such as assisted migration, which is considered an efficient approach to addressing the risk of maladaptation of forest tree populations under climate change (Pedlar et al. 2012). Assisted migration can be implemented at the population level to sustain forest productivity and at the species level to conserve biodiversity. Within the forestry context, assisted migration is mainly carried out at the population level and is confined to the natural range of species, thereby minimizing the risk from exotic invasions, hybridization with novel species, and the introduction of new diseases (Pedlar et al. 2012).

In Alberta, a fixed-zone, geography-based seed transfer system guides the collection, deployment and transfer of seeds collected from wild stands and seed orchards (AAF 2016). In addition, Alberta is challenged by an ongoing reduction in its timber harvesting land base, combined with decreasing forest productivity driven primarily by climate change (Hogg et al. 2017). To address concerns of climate change risk to local populations, we propose exploring opportunities to combine G&Y models with genecology models. We chose to use URFs for their easiness to identify optimal provenances for planting sites (Wang et al. 2010). In addition, we selected GYPSY (Growth and Yield Projection System), a widely used stand-level model, developed empirically, primarily from permanent sample plot data for pure and mixed-species stands that are generally even-aged (Huang et al. 2009). In GYPSY, top height and SI are the main drivers and building a top height curve is the first step for predicting stand volume yield. White spruce (*Picea glauca* (Moench) Voss) and lodgepole pine are the two major commercial conifer species in Alberta and have extensive field trials associated with them. Therefore, in this study, using Alberta as an example, we made use of height measurements from provenance trials for these two species to: (1) build population-specific climate sensitive top height curves by integrating URFs into top height equations in GYPSY, which we then used to; (2) investigate the effect of climate change on productivity of geographically local populations of white spruce and lodgepole pine; and (3) provide guidance for assisted migration using the modified top height curves.

## Materials and methods

### Data resources

#### Provenance trial data

**Fig. 1:**
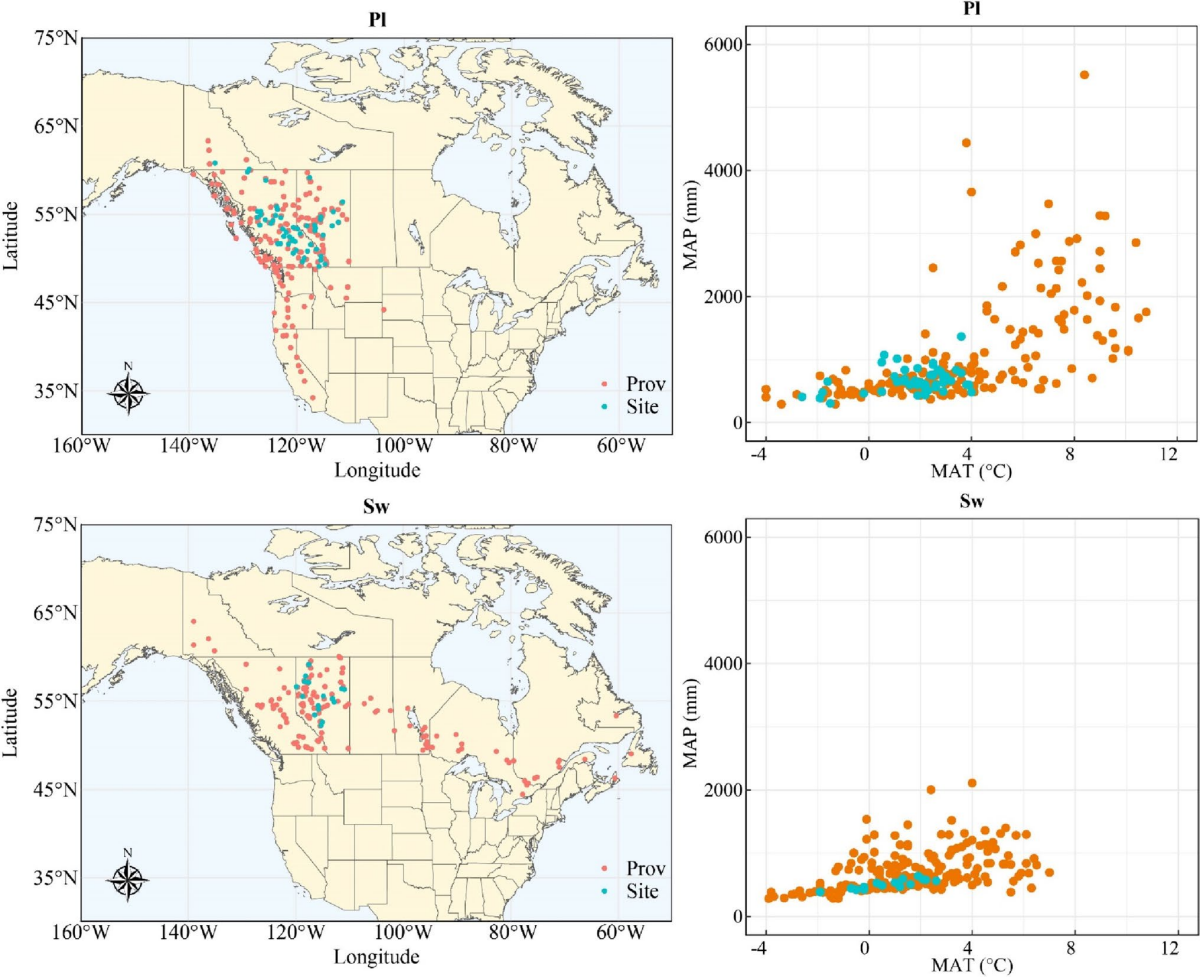
Locations of the test site (Site) and provenance (Prov) of the populations of lodgepole pine (Pl) and white spruce (Sw) trials used in this study. Mean annual temperature (MAT) and mean annual precipitation (MAP) of provenances and test sites across North America

The lodgepole pine trees used in this analysis were from 53 provenance field sites in Alberta, British Columbia, and Yukon containing 194 unique (some populations were used in multiple trials, the same as below for white spruce) wild populations (Fig. 1, Table 1). The ‘Illingworth Trial’, established in 1974 with 140 range-wide lodgepole pine populations tested at 60 test sites (137 populations in 41 test sites were available for this analysis) (Ying et al. 1984). Within each Illingworth site, 60 populations were tested in a randomized complete block design in which each population was tested in each of two blocks in a 3 × 3 tree square with 2.5 m spacing. The remaining 12 lodgepole pine sites in Alberta contained 13–39 populations with five-tree row plots in each of five to nine randomized complete blocks.

**Table 1:** Summary of data sources, by species, trial series code, number (No.) of sites, region, number of populations (popns), establishment (Estab.) year and age of height measurements, used for analysis in this study

Species	Trial series code	No. of sites	Region	No. of popns	Estab. year	Age of height measurements
Lodgepole pine	G134	8	AB	39	1985–1990	10, 15, 20, 25
	Berland 3	1	AB	14	1981	3, 6, 9, 12, 15
	Berland 5	1	AB	14	1980	4, 7, 10, 13, 16
	Marlboro 7	1	AB	16	1979	4, 7, 10, 13, 16
	Embarrass	1	AB	13	1980	4, 7, 10, 13, 16
	Illingworth	41	WNA	137	1974	32
White spruce	G103	10	AB	43	1981–1983	12, 15, 18, 21, 24, 27, 32
	G276	3	AB	21	1993&1994	10, 15, 18, 25
	G277	1	AB	39	1993	7, 10, 15, 18
	G366	1	AB	34	2005	3, 7, 11
	EP 670.71.12	2	AB	119	2005	3, 6, 10, 16

EP 670.71.12; Interior Spruce Climate Change/Genecology Trial

AB; Alberta

WNA; Western North America

White spruce trees used in this analysis were from 17 sites installed in Alberta, with 176 unique wild populations tested (Fig. 1, Table 1). In the ‘Interior Spruce Climate Change/Genecology Trial’, each test site employed an incomplete block design with 119 populations grouped into one of 16 blocks, within which each population was tested in a four-tree row-plot. All remaining test sites contained 21–43 populations of white spruce established in a randomized complete block design containing five to eight blocks, with each population represented in 5–9-tree-row-plots in each block.

#### Climate data

ClimateBC V7.10 (https://climatebc.ca/), ClimateAB v3.21 (https://sites.ualberta.ca/~ahamann/data/climateab.html) and ClimateNA V7.10 (http://climatena.ca/) were used to generate values of 22 annual climate variables for the provenances and test sites located within British Columbia, Alberta, and other areas in western North America, respectively (Wang et al. 2012, 2014) (Table 2). Climate variables without direct biological interpretation were removed (i.e., degree-days below or above 18℃). To reduce the number of independent climate variables to a more manageable number, highly correlated climate variable pairs (r > 0.9) were identified for provenances and sites respectively, and one variable from each pair was excluded, leaving 17 out of 22 site climate variables and 14 out of 22 provenance climate variables for lodgepole pine, and 11 out of 22 site and provenance climate variables for white spruce.

**Table 2:** Annual climate variables, including abbreviation, description and units, used to develop Universal Response Functions (URFs) to assess local adaptation in this analysis

Variable abbreviation	Climate variable description	Unit
AHM	Annual heat-moisture index	℃ mm^−1^
CMD	Hargreaves climatic moisture deficit	Mm
DD_0	Degree-days below 0 ℃	℃ days
DD5	Degree-days above 5 ℃	℃ days
Eref	Hargreaves reference evaporation	mm
EMT	Extreme minimum temperature over 30 years	℃
EXT	Extreme maximum temperature over 30 years	℃
FFP	Frost-free period	day
NFFP	The number of frost-free days	days
bFFP	The day of the year on which FFP begins	day
eFFP	The day of the year on which FFP ends	day
MAP	Mean annual precipitation	mm
MAT	Mean annual temperature	℃
MCMT	Mean coldest month temperature	℃
MWMT	mean warmest month temperature	℃
MAR	mean annual solar radiation	MJ m^−2^ d^−1^
MSP	Mean summer precipitation	Mm
CMI	Hogg’s climate moisture index	Mm
PAS	Precipitation as snow	Mm
RH	Mean annual relative humidity	%
SHM	Summer heat-moisture index	℃ mm^−1^
TD	Temperature difference (continentality; MWMT-MCMT)	℃

#### Geographic data

Digital shape files for the natural range of white spruce and lodgepole pine (https://web.archive.org/web/20170127093428/ and https://gec.cr.usgs.gov/data/little/) were used to illustrate provenance locations.

### Data analysis

Competing vegetation was periodically removed at all sites so that growth of the test trees was primarily a reflection of site climate, edaphic factors, and genetics. Consequently, we assume that each population was able to express its growth potential at each site. Therefore, we used population mean height at each site as the top height after excluding unhealthy trees marked as dead, crooked, browsed by animals, or showing signs of disease. All analyses were done in software *R* 4.2.3 (R Core Team 2018).

#### Universal Response Functions (URFs)

We first estimated population top height at each age and at each site using a linear mixed effect model, with population as a fixed effect and block as a random effect. Given the difficulty of addressing different measurement ages across trials, we then converted the height of each population tested at each site to the common ages of 16 for white spruce and 32 for lodgepole pine using the top height equations from GYPSY (Eqs. 1 and 2) (Huang et al. 2009). The height measurement from the age closest to the common age was identified and converted to height at the common age for those sites where height at the common age was not recorded. The original height was retained for sites with measurements taken at the common age.

1 $$\begin{aligned} & HT_{i.Sw} \\ & \quad = SI*\left( {1 + \exp \left( {b_{1} + b_{2} \sqrt {\ln \left( {1 + 50^{2} } \right)} + b_{3} \left[ {\ln \left( {SI} \right)} \right]^{2} + b_{4} \sqrt {50} } \right)} \right)/\left( {1 + \exp \left( {b_{1} + b_{2} \sqrt {\ln \left( {1 + totage_{i}^{2} } \right)} + b_{3} \left[ {\ln \left( {SI} \right)} \right]^{2} + b_{4} \sqrt {50} } \right)} \right) \\ \end{aligned}$$

2 $$\begin{aligned} & HT_{i.Pl} \\ & \quad = SI*\left( {1 + \exp \left( {f_{1} + f_{2} \sqrt {\ln \left( {1 + 50} \right)} + f_{3} \left( {{\text{ln}}\left( {SI} \right)} \right) + f_{4} \sqrt {50} } \right)} \right)/\left( {1 + \exp \left( {f_{1} + f_{2} \sqrt {\ln \left( {1 + totage_{i} } \right)} + f_{3} \left( {{\text{ln}}\left( {SI} \right)} \right) + f_{4} \sqrt {50} } \right)} \right) \\ \end{aligned}$$

where $HT_{i}$ is the top height (m) at a given total age $i$ for white spruce ($HT_{i.Sw}$) and lodgepole pine ($HT_{i.Pl}$); $SI$ is totage-based site index (top height at 50 years total age) assuming no climate change; $totage_{i}$ = total age i from the point of germination; 50 in equations refer to total age 50 and is a constant; $b_{1} , b_{2} , b_{3} , b_{4} ,f_{1} , f_{2} , f_{3} , f_{4}$ = constants with no biological interpretation.

Next, for each species, we built URFs (Eq. 3) to predict population top heights at the common age using estimated population top height (from Eqs. 1 and 2) and various combinations of retained site and provenance climate (Sect. “Climate data”) as independent variables. Among the retained site and provenance climate variables, we systematically examined combinations ranging from one provenance and one site climate variable to three provenance and three site climate variables. This analysis resulted in the identification of 390,677 candidate URFs for lodgepole pine and 53,361 candidate URFs for white spruce distributed across nine categories of climate variable combinations (Table 3). We selected the URF that had the largest *R*^2^ within each category of climate variable combination. A tenfold cross-validation was applied to the retained candidate URFs for each category of climate variable combination using prediction efficiency (EF, Eq. 4) and residual means square error of prediction (RMSEP, Eq. 5) as the validation metrics. Finally, the candidate URF with the lowest RMSEP was selected as the final model for each species.

3 $$HT_{jt} = exp\left( {a_{1} *x_{j} + a_{2} *x_{t} + a_{3} *x_{j}^{2} + a_{4} *x_{t}^{2} + a_{5} *x_{j} *x_{t} } \right) + \varepsilon_{jt}$$

4 $$EF = 1 - \left[ {\sum \left( {Y_{k} - \widehat{{Y_{k} }}} \right)^{2} /\sum \left( {Y_{k} - \overline{Y}} \right)^{2} } \right]$$

5 $$RMSEP = \sqrt {\mathop \sum \limits_{k = 1}^{n} \left( {Y_{k} - \hat{Y}_{k} } \right)^{2} /n}$$

where $HT_{jt}$ is the estimated top height for the $j$ th population estimated at test site $t$; $x_{j}$ is the provenance climate variable for the $j$ th population (averaged during 1961–1990 normal period); $x_{t}$ is the climate variable for $t$ th test site (averaged from planting year until measurement age); $a_{1}$–$a_{5}$ are coefficients to be determined; $n$ is the number of observed values; $Y_{k}$ is the $k$ th observed value; $\widehat{{Y_{k} }}$ is the $k$ th predicted value; $\overline{Y}$ is the average of the observed values.

**Table 3:** Results of *R*^2^ (coefficient of determination), AIC (Akaike information criterion), number (No.) of test sites and population (popn) residual mean square error of predictions (RMSEP), and prediction efficiency (EF) for fitting Universal Response Functions (URFs) with various combinations of site and provenance climate variables to population top height of lodgepole pine at age 32 and white spruce at age 16

Species	Climate variable	*R*^2^	AIC	No. site	No. popn	RMSEP (m, tenfold)	EF (tenfold)
Lodgepole pine	TD.p; MAT.s	0.395	13,474.6	53	194	2.29	0.463
	TD.p; MCMT.s; DD5.s	0.453	13,208.5	53	194	2.15	0.527
	TD.p; TD.s; eFFP.s; EXT.s	0.478	13,083.1	53	194	2.06	0.564
	MAT.p; TD.p; MAT.s	0.419	13,369.9	53	194	2.21	0.498
	TD.p; CMD.p; MCMT.p; DD5.s	0.485	13,050.3	53	194	2.06	0.567
	TD.p; Eref.p; MCMT.s; eFFP.s; EXT.s	0.524	12,855.3	53	194	1.98	0.598
	MAT.p; TD.p; CMD.p; MAT.s	0.457	13,194.2	53	194	2.17	0.516
	MAT.p; TD.p; CMD.p; MCMT.s; DD5.s	0.503	12,962.3	53	194	1.99	0.596
	**MAT.p; TD.p; CMD.p; MCMT.s; DD5.s; PAS.s**	**0.527**	**12,833.0**	**53**	**194**	**1.88**	**0.637**
White spruce	MCMT.p; MAP.s	0.603	1565.8	17	176	0.96	0.447
	MCMT.p; MAP.s; FFP.s	0.634	1520.8	17	176	0.91	0.502
	MAT.p; MAP.s; SHM.s; FFP.s	0.593	1593.1	17	176	0.87	0.554
	MCMT.p; AHM.p; MAP.s	0.622	1539.6	17	176	0.95	0.467
	MCMT.p; AHM.p; MAP.s; FFP.s	0.654	1490.5	17	176	0.90	0.519
	AHM.p; Eref.p; MAP.s; SHM.s; FFP.s	0.687	1438.1	17	176	0.75	0.663
	MCMT.p; Eref.p; RH.p;MAP.s	0.649	1500.0	17	176	0.91	0.511
	MCMT.p; MSP.p; SHM.p; MAP.s; FFP.s	0.672	1462.5	17	176	0.88	0.542
	**AHM.p; NFFD.p; Eref.p; MAP.s; SHM.s; FFP.s**	**0.700**	**1416.1**	**17**	**176**	**0.75**	**0.673**

Suffix p and s refer to provenance and site; full names of climate variables are listed in Table 2; bold font indicates the selected URFs

#### Merging URFs into top height curves

Following the method of O'Neill and Nigh (2011) and Nigh (2014), we merged URFs instead of Pooled Transfer Functions into top height curves in GYPSY. The final population-specific climate sensitive top height curves were integrated annual height increment derived from climate non-sensitive top height curves built on different plantation climates at corresponding ages for different populations.

In the first step, we estimated top height at age 32 for lodgepole pine and at age 16 for white spruce using URFs (Eq. 3). The purpose of this step was to calculate an initial value for estimating population height at different ages and under different climates. The plantation climates used in URFs were averaged from planting year to age i in the final population-specific climate sensitive top height curves (e.g., age i ranges from 1 to 80, corresponding climates were from year 1 to i, therefore 80 top height values for age i were derived from URFs for a provenance-plantation combination). Climate values during the 1961–1990 normal period were used to represent the population historic climate, that is, the climate to which the populations are expected to be best adapted (Wang et al. 2012, 2014).

In the second step, top height interpreted from URFs was substituted into top height equations (Eqs. 1–2) to solve for the corresponding SI, as GYPSY requires SI to be determined before generating top height curves.

In the third step, the solved SI was used to generate a set of climate non-sensitive top height curves for the given provenance-plantation climate combination (e.g., 80 climate non-sensitive top height curves). The top height curves generated in the third step represent 80 different static climates.

**Fig. 2:**
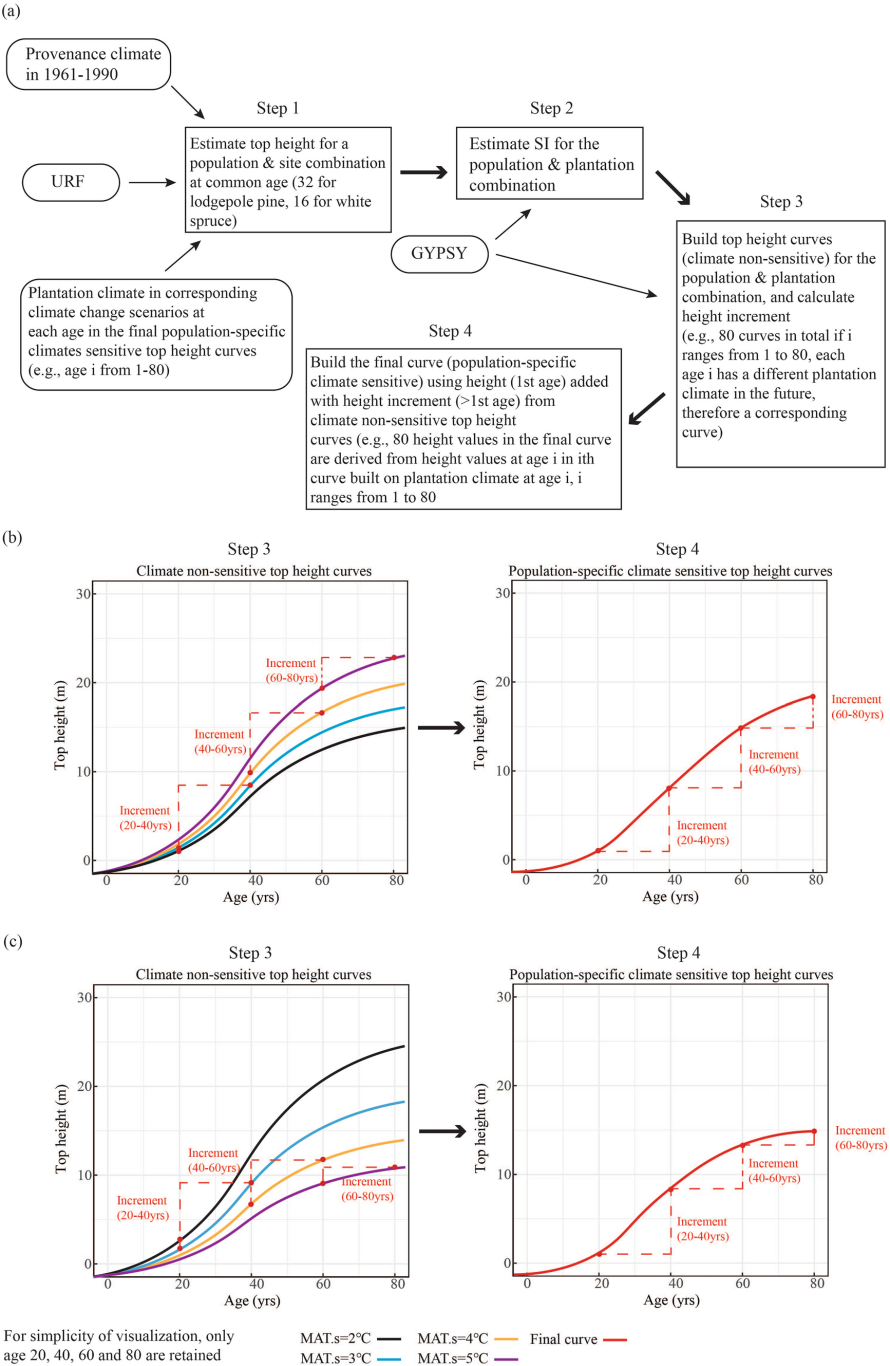
Flow chart for building a population-specific climate sensitive top height curve (a); population-specific climate sensitive and climate non-sensitive top height curves in the case of top height increasing with site mean annual temperature (b); and top height decreasing with site mean annual temperature (c); under climate change scenarios; URF is Universal Response Function; GYPSY is Growth and Yield Projection System; MAT.s is site mean annual temperature at age i

In the fourth step, we built a population-specific climate sensitive top height curve using top height values extracted from the set of climate non-sensitive top height curves generated in the third step. Top height value at age one was retained, while for each climate non-sensitive curve built on plantation climate at age i (i > 1), annual height increment at age i was extracted and added to top height values calculated at age i-1. These extracted single-age height values were assembled as the final top height curve for the selected population. The same procedure can be repeated for different populations with various provenance climates to produce population-specific climate sensitive top height curves. The future climate change scenario is described below in Sect. “Application of the population-specific climate sensitive top height curves”. The flow chart is shown in Fig. 2, where four climate non-sensitive top height curves instead of 80 are used for a simplified visualization.

**Fig. 3:**
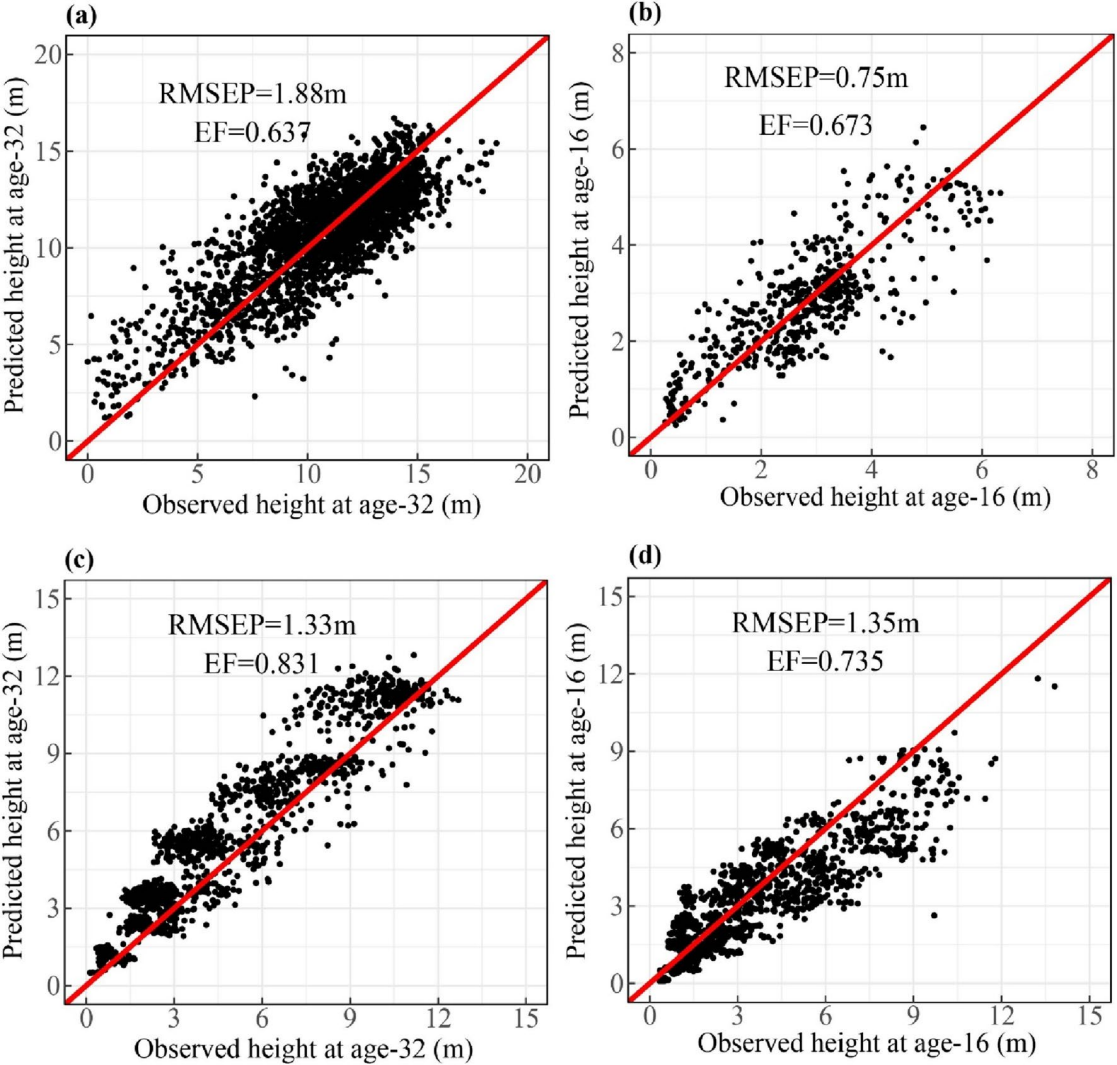
Results from the tenfold cross validation for the selected Universal Response Function (URF) for height estimated at age-32 for lodgepole pine (a); and height estimated at age-16 for white spruce (b); results from the tenfold cross validation for the selected population-specific climate sensitive top height curves for Pl (c); and Sw (d); RMSEP is residual means square error of prediction; EF is prediction efficiency

A tenfold validation was applied to the final population-specific climate sensitive top height-age curves using EF (Eq. 4) and RMSEP (Eq. 5) as validation metrics where top height values from other measurement ages were taken as the validation sets (Fig. 3).

#### Application of the population-specific climate sensitive top height curves

In the subsequent analysis, future climate values, including annual and normal period means, were obtained from an ensemble climate projection from 13 General Circulation Models and were included in population-specific climate sensitive top height curves. Shared Socioeconomic Pathway (SSP)245 and SSP585 were selected as the climate change scenarios. SSP245 indicates a climate change scenario described in CMIP 6 (Coupled Model Intercomparison Project Phase 6), in which social, economic, and technological trends do not shift markedly from historical patterns, with an additional radiative forcing of 4.5 W/m^2^ by the year 2100 (Eyring et al. 2016). SSP585 indicates a scenario of unconstrained economic growth and high energy demand for fossil fuels, with an additional radiative forcing of 8.5 W/m^2^ by the year 2100 (Eyring et al. 2016). Although there are other scenarios, we used these two for comparison as they represent the current trend and the worst situation of global warming.

##### Predicting climate change impacts on local populations

**Fig. 4:**
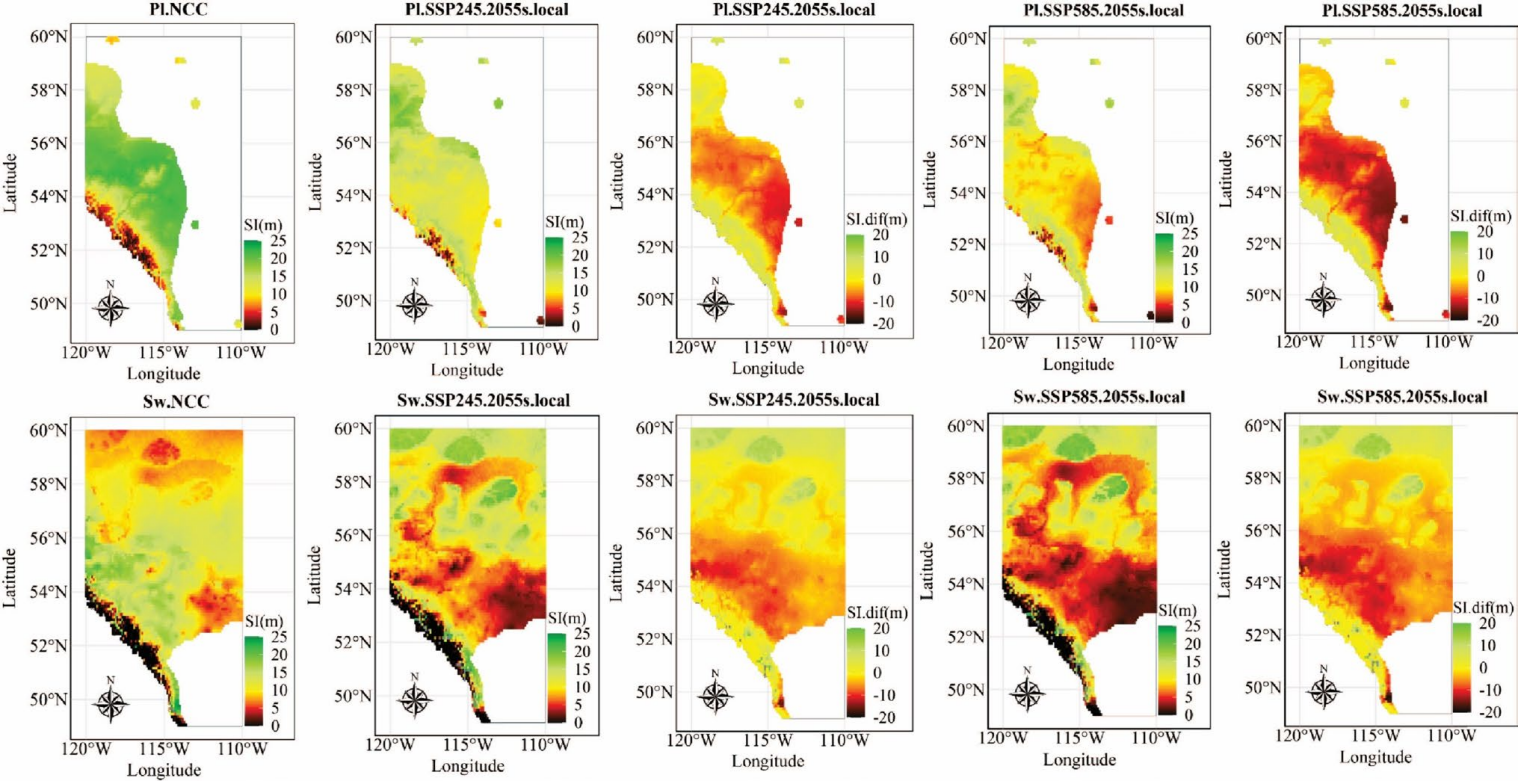
Site index (SI, age 50) and site index difference (SI.dif) for the local populations of lodgepole pine (Pl) and white spruce (Sw) under no climate change (NCC), Shared Socioeconomic Pathways (SSP)245 and SSP585 in 2055s (averaged from 2041–2070) within each species’ current natural distribution in Alberta

**Fig. 5:**
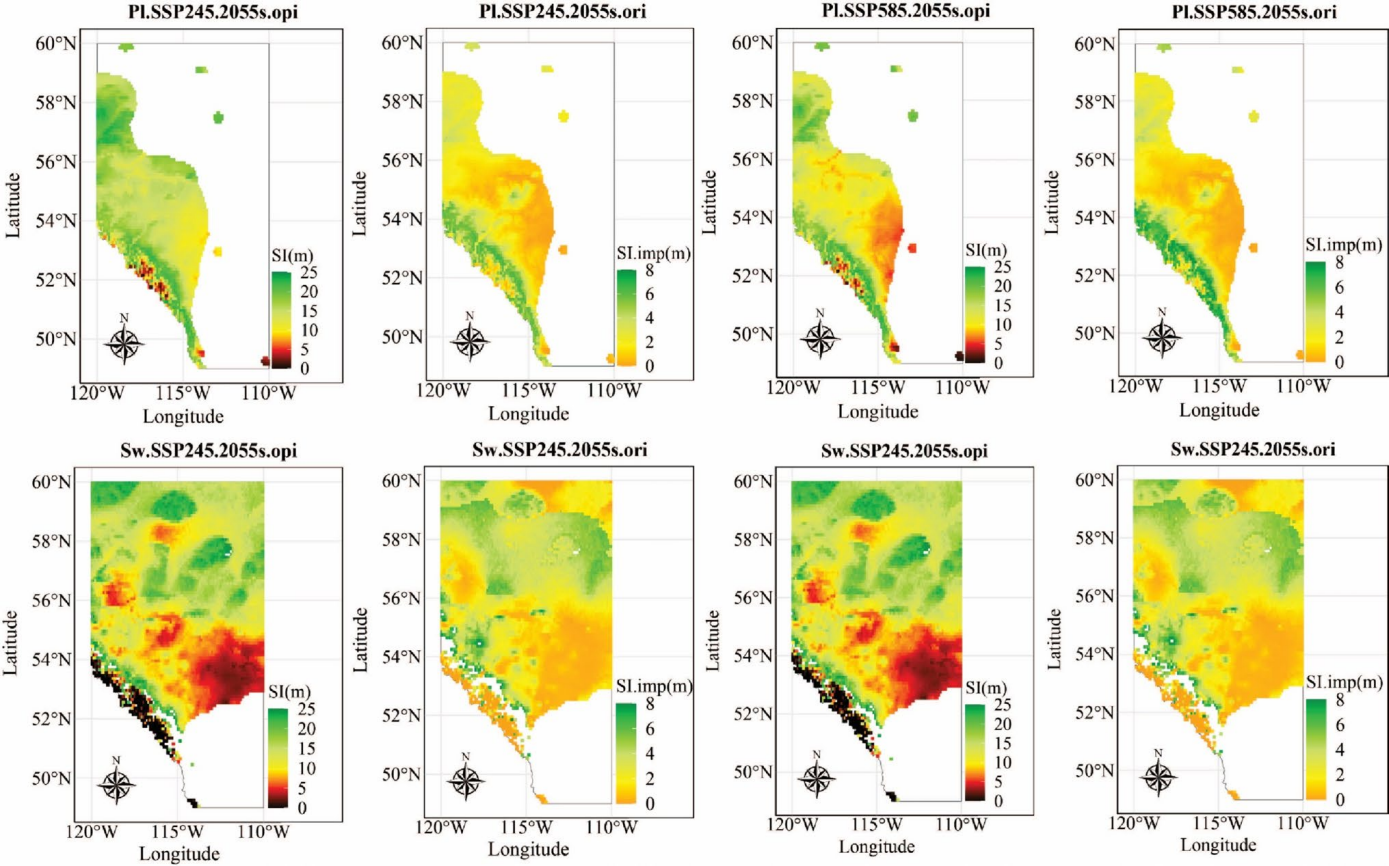
Site index (SI, age 50) and site index improvement compared to local populations (SI.imp) for the optimal populations (.opi) of lodgepole pine (Pl) and white spruce (Sw) under Shared Socioeconomic Pathways (SSP)245 and SSP585 in 2055s (averaged from 2041–2070) within species natural range in Alberta

The provincial map for SI was generated using population-specific climate sensitive top height curves and a grid set covering Alberta with a resolution of 0.5 °C (Figs. 4 and 5). To simplify the visualization, we selected 2055-representing the climate normal for 2041–2070—as the year when total height reached age-50, and the corresponding 30-year climate average was used as site climate under which trees will be growing in the future. The climate average in normal period 1961–1990 was used as the provenance climate.

The SI maps generated demonstrate the effects of climate change at age-50 (Figs. 4 and 5). To demonstrate the cumulative effects of climate change on yield curves, we selected two test sites and built top height curves for each species, including site G346a (MAT = -0.7 °C) and WHIT (MAT = 3.9 °C) for lodgepole pine (Pl) and site G366a (MAT = -1.9 °C) and site G354a (MAT = 2.6 °C) for white spruce (Sw). The selected sites represented cold and warm locations and were selected to demonstrate the effect of site temperature on top height. Large inter-annual variation in climate values resulted in fluctuations in top height curves. Therefore, to capture the long-term trend of climate change and to produce smooth top height curves, a log regression was fitted to the lifespan averaged climate for each age. The estimated climate values were then used in the population-specific climate sensitive top height curves.

##### Identifying effects of selecting optimal populations

Using the population-specific climate sensitive top height curves, we identified the provenance climate in the normal period 1961–1990 of the population expected to generate the tallest trees at age-50 (year 2055) at the provincial scale to help optimize seed source selection considering climate change. To achieve this goal, for each grid point from the provincial map, we scanned the natural range within the province using a grid with an adjustable resolution (e.g. 0.5° or 1000 random points, while only 100 random points were selected for calculation efficiency) and applied the population-specific climate sensitive top height curves to the 100 grid points to identify the climate of the population expected to yield the greatest growth.

A visual inspection of climate at the provincial scale revealed that the mountainous regions of southwestern Alberta were expected to undergo significant changes in precipitation due to climate change (Fig. 7 in the Appendix), surpassing the precipitation observed in the current provenance trials. These regions closely matched locations where the SI exceeded 25 m when considering optimal populations under the climate change scenario (Figs. 4, 5). To minimize the risk associated with extrapolation, we decided to exclude sites with an SI greater than 25 m.

The procedure of optimal population selection was also applied to the selected four test sites identified in the previous section.

## Results

### Universal Response Function (URF)

The final URF model for lodgepole pine (*R*^2^ = 0.527) contained three provenance climate variables (mean coldest month temperature, continentality and Hargreaves climatic moisture deficit) and three site climate variables (mean coldest month temperature, degree-days above 5 ℃, and precipitation as snow), while the final URF for white spruce (*R*^2^ = 0.680) contained three provenance climate variables (annual heat-moisture index, the number of frost-free days, and Hargreaves reference evaporation) and three site climate variables (mean annual precipitation, summer heat moisture, and frost-free period) (Tables 3 and 4 in the Appendix). In the tenfold validation, the selected URF for lodgepole pine had a RMSEP equal to 1.88 m and EF equal to 0.637, while the selected URF for white spruce had a RMSEP equal to 0.75 m and EF equal to 0.673 (Table 3 and Fig. 3).

### Population-specific climate-sensitive top height curves

#### Validation of population-specific climate sensitive top height curves

During the tenfold cross-validation process, in which height measurements from other ages were used as validation sets, the population-specific climate sensitive top height curves yielded high prediction EF values. Specifically, lodgepole pine achieved an EF value of 0.831, while white spruce achieved an EF value of 0.735 (Fig. 3).

#### Predicting climate change impacts on local populations

**Fig. 6:**
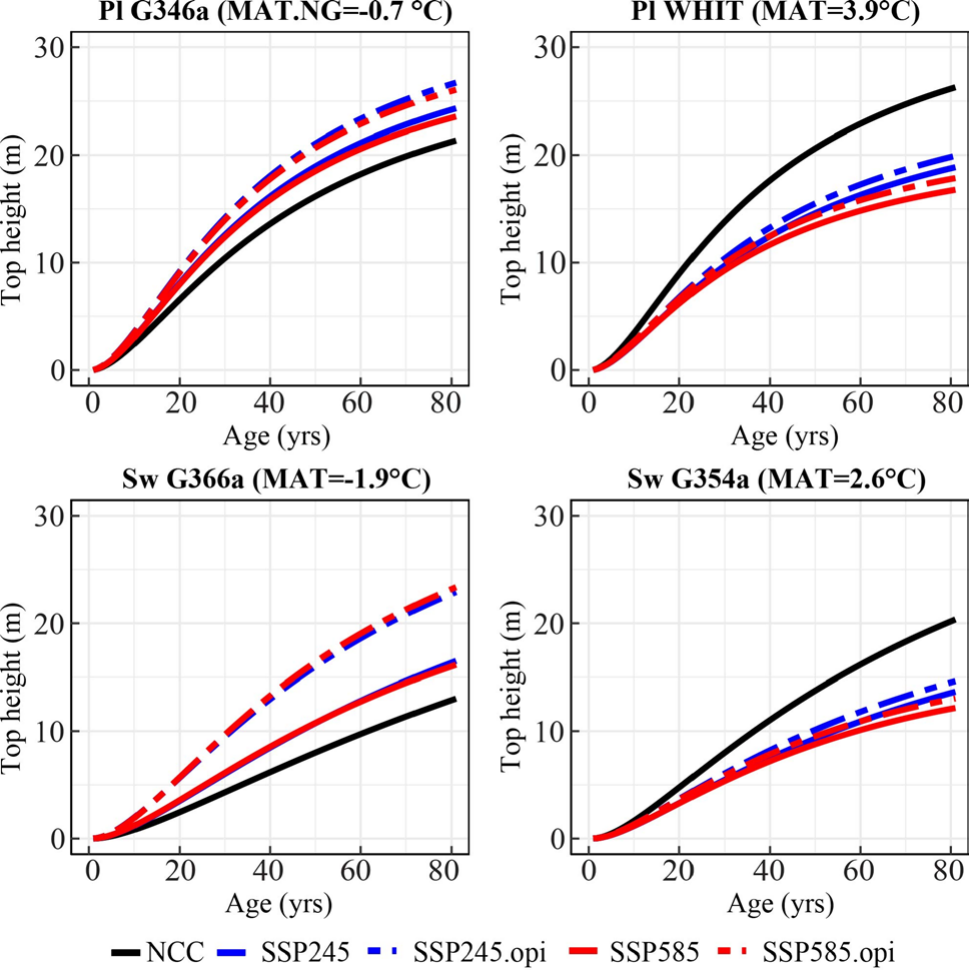
Case study of population-specific climate sensitive top height curves for lodgepole pine (Pl) growing at site G346a, site WHIT, and white spruce (Sw) at site G366a, G354a under no climate change (NCC), Shared Socioeconomical Pathway (SSP) 245 and SSP 585; optimal population (.opi) is selected based on grid scanning (100 random points) that cover the natural range within Alberta

**Fig. 7:**
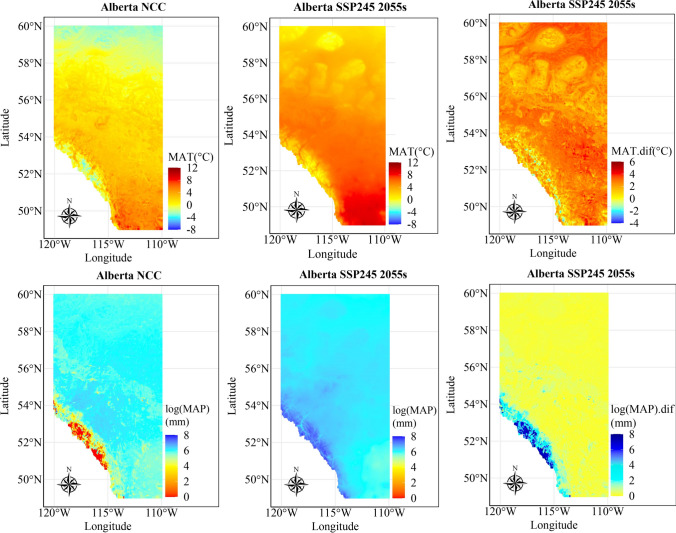
Mean annual temperature (MAT), log mean annual precipitation (MAP) and their change (dif) in Alberta under no climate change (NCC) and under Shared Socioeconomic Pathway (SSP) 245 in the 2055s (averaged over 2041–2070)

Application of the population-specific climate-sensitive top height curves at the provincial scale revealed strong impacts of climate change on forest productivity. The anticipated climate change was projected to have negative impacts on site index (SI) of lodgepole pine in central Alberta, while a positive impact on SI was projected in mountainous areas located in the southwest and part of northern Alberta (Fig. 4). In the case of white spruce, a slight increase in SI was projected for the mountain area in southwestern Alberta and part of northern Alberta, while a decrease was projected in most central areas. Overall, the negative impacts from climate change under SSP585 appeared stronger compared to SSP245 for both species. The case study conducted on the selected test sites revealed that the anticipated climate change may have mixed effects on forest productivity. Specifically, in site G346a for lodgepole pine and site G366a for white spruce, the changing climate was projected to improve height growth. While in site WHIT for lodgepole pine and site G354a for white spruce, the changing climate was anticipated to negatively impact top height yield (Fig. 6). In the selected sites, height growth under SSP585 was slightly lower than SSP245, indicating a negative impact of further increasing temperature regardless of original site temperature.

#### Identifying effects of selecting optimal populations

Regardless of species, the selection of optimal populations was projected to maintain and even enhance the SI in most areas (Fig. 5). In the test sites G346a and G366a, selecting the optimal population from within the natural range in Alberta had the potential to further enhance the SI. In site WHIT and G354a, the selected optimal population could mitigate the projected negative impacts (Fig. 6).

## Discussion

### Model performance

Using provenance URFs developed from comprehensive sets of provenance data, we were able to modify top height curves in the G&Y models (GYPSY) to simulate the impacts of climate change on forest productivity. With the objective of being able to predict climate change impacts, performance of the final population-specific climate sensitive top height curves in this study was assessed through a tenfold cross validation. The tenfold cross-validation results indicated that the population-specific climate sensitive top height curves had good predictive accuracy and predictive power, with an EF equal to 0.831 for lodgepole pine and 0.735 for white spruce.

Both temperature and precipitation-related variables played important roles in the final population-specific climate sensitive top height curves (Table 3). Temperature-related variables are normally the most important climate factors influencing geographic patterns of provenance performance for tree species (Leites et al. 2012a, 2012b). Conifer species exhibit different growth rates, phenology events, and metabolic activity that are mainly associated with temperature (Guo et al. 2020). The low temperature in winter and early spring are generally considered as an important factor limiting survival and growth for conifers due to its potential frost damage (Pearce 2001). Freezing temperatures can also lead to dehydration due to extracellular freezing of water (Jeffree et al. 1987). In contrast, increasing temperature may result in a longer growing season and higher carbon sequestration assuming water availability is not a limitation (Keenan et al. 2014). Precipitation is another important climate factor influencing tree growth and interacts with changing temperature. Under increasing temperature, the potential damage could be caused by limited water availability as it may reduce photosynthesis, and therefore inhibit growth and even lead to increased mortality (McDowell et al. 2016; Hogg et al. 2017). Under global warming, the challenge of rising temperature appears to outweigh its benefit, given that most areas in Alberta are predicted to have limited decreasing precipitation, as suggested by our results.

Integrating URFs into top height curves offers a comprehensive strategy that harnesses the strengths of both genecology models and G&Y models. This integration presents a promising opportunity to enhance existing climate-sensitive methodologies for the selection of optimal populations for plantation sites. Prior research merging climate variables into G&Y (e.g., height increment curves, diameter increment curves) used data from permanent sample plots (PSPs) (Yang et al. 2019), which often overlooked population effects and their interaction with the environment. In contrast, merging Pooled Transfer Functions into G&Y curves (O'Neill and Nigh 2011; Nigh 2014) using data from provenance trials integrates population’s variation to climate, thereby furnishing valuable insights for the selection of suitable seed sources. It is worth noting that by standardizing height at each site, differences in productivity among sites were removed in Pooled Transfer Functions. Without a site climate variable as input, variation in site productivity cannot be determined. To address this, supplementary information such as the estimated SI across different locations becomes imperative before implementing a merged G&Y model with Pooled Transfer Functions. Consequently, our study modified top height curves in GYPSY using URFs, which included genetic effects of populations, climate effects, and their interaction. While both URFs and UTFs perform similarly (Zhao and Wang 2023), incorporation of provenance, site climate, and their interactions in URFs, provides a more intuitive approach for identifying the optimal population or plantation site compared to UTFs (O'Neill et al. 2008). This updated climate-sensitive methodology to identify the climate of the most productive seed source could obviate the need to assume that productivity will be optimized by matching historic population climate and future climate of plantations at ¼ of the rotation age (O’Neill et al. 2008, 2017; Ukrainetz et al. 2011).

### The effects of climate change on forest productivity for different populations

The effects of climate change on forest productivity vary for different populations under different provenance and site climates, both increasing and decreasing tree growth were observed. Previous studies demonstrate that growth response of forest populations to climate conditions differs in western Canada, where the variation of forest productivity has shown a strong association with climate conditions (O'Neill et al. 2008; Wang et al. 2010; Leites et al. 2012a, 2012b; Hogg et al. 2017). For instance, in British Columbia, significant reductions in stand yield for lodgepole pine were predicted in most areas based on provenance trial data, except for an increase in stand yield observed in northern regions (O'Neill et al. 2008). In Alberta, by using stem analyses on trees from natural stands, a decreasing trend of aboveground biomass for white spruce was reported throughout most areas of the province, except in northern areas where an increase in aboveground biomass was observed (Hogg et al. 2017). Our study indicated similar patterns, in which decreased productivity was predicted in central Alberta, while increased productivity was projected in the southwestern mountain areas and some northern areas. In Alberta, the southern mountain areas are colder than the central regions and are therefore more likely to be the leading edge for species migration under climate change. In addition, our findings suggest that under the high-emission SSP585 scenario, tree height is expected to decrease to a larger extent at the provincial scale compared to SSP245 scenario. This trend could be due to the further increased stress from higher temperatures and relatively limited water availability, affecting tree growth. The differential impact between scenarios highlights the significance of emission levels on forest ecosystems and the potential for more severe ecological consequences under higher emission pathways.

The phenomenon of climate change impacting forest productivity manifests consistently across similar latitudes worldwide. Relevant research projects across Europe show that forest productivity increases in northern Europe, varies in central Europe, and declines in southern Europe (Reyer et al. 2014). Temperature and drought related tree mortality was also reported across Europe, while a warmer winter is found to compensate for the mortality (Neumann et al. 2017; Senf et al. 2020). Other research indicates that climate warming is expected to significantly alter forest distribution and stand heights in central Siberia by the 2080s, where stand heights are predicted to rise, with highly productive forests expanding northward at the expense of less forest habitat in the southern border (Tchebakova et al. 2016). These patterns are similar to our findings given that the northern area with lower temperature is on the leading edge of species migration.

### Implications for forest management

The population-specific climate-sensitive top height curves developed here integrate the effects of provenance climate, site climate and their interaction, alongside cumulative climate effects on tree growth, may therefore provide an improved tool for guiding assisted migration. Alberta’s seed transfer system and almost all others worldwide (O’Neill et al. 2017) constrain transfer of reforestation seed to within the fixed seed zone (applied to wild stand seed sources) or breeding region (applied to orchard seed sources) from which a seed source originates (AAF 2016). The findings of this study, however, challenge this paradigm by suggesting that mitigating climate change impacts may require movement of seed outside its seed zone of origin. Populations that are currently well adapted to prevailing climates may face challenges in the future due to changing climatic conditions (Wang et al. 2014; Hogg et al. 2017). Therefore, alternative seed transfer systems which are flexible enough in responding to climate change are urgently needed. Combining URFs (Wang et al. 2010) with GYPSY (Huang et al. 2009) enables the interaction between genotype and environment to be further delineated and explored in the long-term, and therefore provides valuable insight for assisted migration practices. Although we used Alberta as an example to demonstrate the application of the population-specific climate sensitive top height curves, it is important to note that this approach is not restricted to a particular geographic area. The adaptability of population-specific climate sensitive top height curves extends to various tree species by incorporating corresponding G&Y models relevant to distinct regions and corresponding provenance trials.

The most productive (optimal) population for a given site was often found outside the seed zone within which the plantation was located, as indicated by the difference between optimal and local populations. Previous studies have reported that ecologically optimum climate (competitively exclusive) of a population is often not equal to its physiological optimum climate (competitively excluded) (Leites et al. 2012a; Rehfeldt et al. 2018). This finding of non-local optimality also supports the idea of ‘gene swamping’ (Aitken et al. 2008), in which there exists a net flow of pollen from the center of a species’ distribution where populations are dense (large numbers of trees/ha) toward peripheral populations where densities are lower. This net flow of pollen can result in an evolutionary lag among peripheral populations, such that their adaptation is more like those from the central populations than one would expect from their climate (Aitken et al. 2008). This difference between ‘ecological optimum’ and ‘physiological optimum’ combined with expected exacerbation of the evolutionary lag under climate change, further indicates the necessity of matching populations with the climate to which they are best adapted to maintain forest productivity.

By examining population height growth in provenance trials where populations are pushed to their physiological limit, and inter-species competition, physical barriers, historical events, and human impact are avoided (Aitken et al. 2008; Zhao and Wang 2023), we were able to predict productivity within the fundamental niche rather than the realized niche. By predicting height growth in the fundamental niche, we aimed to capture the potential productivity of populations under plantation conditions, where such barriers are minimal (Ying and Yanchuk 2006). Assisted migration involves relocating populations to ensure that anticipated climate conditions are matched with the optimal populations (Pedlar et al. 2012). Therefore, the prediction of productivity within the fundamental niche aligns well with the objectives and principles of assisted migration.

## Conclusion

The implementation of assisted migration as a climate change adaptation strategy can be bolstered by a robust quantitative method. Taking advantage of existing provenance trials, we introduced a novel approach to develop population-specific climate sensitive top height curves, which can be used to modify growth and yield models. This novel approach addresses the limitations of current assisted migration strategies by incorporating the cumulative effects of climate on tree growth, combined with population variation and climate interactions, and therefore provides a new option to forest managers faced with managing forests under a changing climate.

## Data Availability

Data is available upon request.

## Appendix

See Fig. 7 and Table 4.

**Table 4:** Parameter estimates of the selected Universal Response Functions (URFs) for lodgepole pine and white spruce

Variable	Parameter estimate	*p*_value	Partial *R*^2^	Variable	Parameter estimate	*p*_value	Partial *R*^2^
Lodgepole pine				White spruce			
Intercept	− 2.853000	< 0.001		Intercept	− 59.360000	< 0.001	
MAT.p^2^	− 0.005774	< 0.001	0.069	AHM.p	− 0.710200	< 0.001	0.039
TD.p	0.048300	< 0.001	0.005	AHM.p^2^	− 0.001835	< 0.001	0.019
TD.p^2^	− 0.002111	< 0.001	0.082	NFFD.p	0.025340	< 0.001	0.029
CMD.p^2^	− 0.000003	< 0.001	0.025	NFFD.p^2^	− 0.000102	< 0.001	0.018
MCMT.s	− 0.154400	< 0.001	0.069	Eref.p	0.091070	< 0.001	0.001
MCMT.s^2^	− 0.008370	< 0.001	0.032	Eref.p^2^	− 0.000005	< 0.001	0.006
DD5.s	0.004286	< 0.001	0.085	MAP.s	0.087060	< 0.001	0.232
DD5.s^2^	− 0.000002	< 0.001	0.042	MAP.s^2^	− 0.000036	< 0.001	0.144
PAS.s	0.002537	< 0.001	0.011	SHM.s	0.460300	< 0.001	0.046
PAS.s^2^	− 0.000003	< 0.001	0.009	SHM.s^2^	− 0.001124	< 0.001	0.001
MCMT.s:MAT.p	0.004568	< 0.001	0.001	FFP.s	0.103700	< 0.001	0.002
TD.p:MCMT.s	− 0.003742	< 0.001	0.060	FFP.s^2^	− 0.000812	< 0.001	0.030
CMD.p:MCMT.s	0.000169	< 0.001	0.023	AHM.p:MAP.s	0.001109	< 0.001	0.004
DD5.s:MAT.p	0.000112	< 0.001	0.005	AHM.p:SHM.s	0.008649	< 0.001	0.010
TD.p:DD5.s	0.000042	< 0.001	0.004	AHM.p:FFP.s	− 0.001704	< 0.001	0.003
CMD.p:DD5.s	− 0.000001	< 0.001	0.003	Eref.p:MAP.s	− 0.000116	< 0.001	0.044

p and s refer to provenance and site; full names of each climate variables are listed in Table 4

## Code availability

Code available upon request to corresponding author.

## References

[CR1] AAF (Alberta Agriculture and Forestry). 2016. Alberta forest genetic resource management and conservation standards. Alberta Agriculture and Forestry, Government of Alberta, Edmonton, Alberta.

[CR2] Aitken SN, Yeaman S, Holliday JA, Wang T, Curtis-McLane S (2008) Adaptation, migration or extirpation: climate change outcomes for tree populations. Evol Appl 1:95–111 25567494 10.1111/j.1752-4571.2007.00013.x PMC3352395

[CR3] Eyring V, Bony S, Meehl GA, Senior CA, Stevens B, Stouffer RJ, Taylor KE (2016) Overview of the coupled model intercomparison project phase 6 (CMIP6) experimental design and organization. Geosci Model Dev 9:1937–1958

[CR4] Guo X, Khare S, Silvestro R, Huang J, Sylvain J-D, Delagrange S, Rossi S (2020) Minimum spring temperatures at the provenance origin drive leaf phenology in sugar maple populations. Tree Physiol 40:1639–1647 32705120 10.1093/treephys/tpaa096

[CR5] Hogg EH, Michaelian M, Hook TI, Undershultz ME (2017) Recent climatic drying leads to age-independent growth reductions of white spruce stands in western Canada. Glob Change Biol 23:5297–5308 10.1111/gcb.13795 28636146

[CR6] Huang SM, Meng SX, Yang YX (2009) A Growth and Yield Projection System (GYPSY) for natural and post-harvest stands in Alberta. Forest Management Branch, Edmonton, Alberta, Canada

[CR7] Jeffree C, Read N, Smith J, Dale J (1987) Water droplets and ice deposits in leaf intercellular spaces: redistribution of water during cryofixation for scanning electron microscopy. Planta 172:20–37 24225784 10.1007/BF00403025

[CR8] Keenan TF, Gray J, Friedl MA, Toomey M, Bohrer G, Hollinger DY, Munger JW, O’Keefe J, Schmid HP, Wing IS (2014) Net carbon uptake has increased through warming-induced changes in temperate forest phenology. Nat Clim Chang 4:598–604

[CR9] Leimu R, Fischer M (2008) A meta-analysis of local adaptation in plants. PLoS ONE 3:e4010 19104660 10.1371/journal.pone.0004010 PMC2602971

[CR10] Leites L, Rehfeldt P, Robinson AP, Crookston NL, Jaquish B (2012a) Possibilities and limitations of using historic provenance tests to infer forest speices growth responses to climate change. Nat Resour Model 25:409–433

[CR11] Leites LP, Robinson AP, Rehfeldt GE, Marshall JD, Crookston NL (2012b) Height-growth response to climatic changes differs among populations of Douglas-fir: a novel anlaysis of historic data. Ecol Appl 22:154–165 22471081 10.1890/11-0150.1

[CR12] Malcolm JR, Markham A, Neilson RP, Garaci M (2002) Estimated migration rates under scenarios of global climate change. J Biogeogr 29:835–849

[CR13] McDowell NG, Williams AP, Xu C, Pockman WT, Dickman LT, Sevanto S, Pangle R, Limousin J, Plaut J, Mackay DS, Ogee J, Domec JC, Allen CD, Fisher RA, Jiang X, Muss JD, Breshears DD, Rauscher SA, Koven C (2016) Multi-scale predictions of massive conifer mortality due to chronic temperature rise. Nat Clim Chang 6:295–300

[CR14] Mitchell KJ (1975) Dynamics and simulated yield of Douglas-fir. For Sci Monogr 17:1–39

[CR15] Neumann M, Mues V, Moreno A, Hasenauer H, Seidl R (2017) Climate variability drives recent tree mortality in Europe. Glob Change Biol 23:4788–4797 10.1111/gcb.13724 PMC5633074 28417562

[CR16] Nigh G (2014) Mitigating the effects of climate change on lodgepole pine site height in British Columbia, Canada, with a transfer function. Forestry 87:377–388

[CR17] O’Neill G, Ukrainetz N, Carlson M, Cartwright C, Jaquish B, King J, Krakowski J, Russell J, Stoehr M, Xie C (2008) Assisted migration to address climate change in British Columbia recommendation for interim seed transfer standards

[CR18] O’Neill G, Wang T, Ukrainetz N, Charles L, MacAuley L, Yanchuck AD, Zedel S (2017) A proposed Climate-based Seed Transfer System for British Columbia. Prov. B.C., Victoria, B.C. Tech. Rep. 099*.* www.for.gov.bc.ca/hfd/pubs/Docs/Tr/Tr099.htm

[CR19] O’Neill GA, Nigh G (2011) Linking population genetics and tree height growth models to predict impacts of climate change on forest production. Glob Change Biol 17:3208–3217

[CR20] O’Neill GA, Hamann A, Wang T (2008) Accounting for population variation improves estimates of the impact of climate change on species growth and distribution. J Appl Ecol 45:1040–1049

[CR21] Pearce R (2001) Plant freezing and damage. Ann Bot 87:417–424

[CR22] Pedlar JH, McKenney DW, Aubin I, Beardmore T, Beaulieu J, Iverson L, O’Neill GA, Winder RS, Ste-Marie C (2012) Placing forestry in the assisted migration debate. Bioscience 62:835–842

[CR23] R Core Team (2018) R: a language and environment for statistical computing. R Foundation for Statistical Computing, Vienna, Austria

[CR24] Rehfeldt GE, Leites LP, Joyce DG, Weiskittel AR (2018) Role of population genetics in guiding ecological responses to climate. Glob Change Biol 24:858–868 10.1111/gcb.13883 28862811

[CR25] Reyer C, Lasch-Born P, Suckow F, Gutsch M, Murawski A, Pilz T (2014) Projections of regional changes in forest net primary productivity for different tree species in Europe driven by climate change and carbon dioxide. Ann for Sci 71:211–225

[CR26] Senf C, Buras A, Zang CS, Rammig A, Seidl R (2020) Excess forest mortality is consistently linked to drought across Europe. Nat Commun 11:6200 33273460 10.1038/s41467-020-19924-1 PMC7713373

[CR27] Skovsgaard JP, Vanclay JK (2008) Forest site productivity: a review of the evolution of dendrometric concepts for even-aged stands. Forestry 81:13–31

[CR28] Sperlich D, Nadal-Sala D, Gracia C, Kreuzwieser K, Hanewinkel M, Yousefpour R (2020) Gains or losses in forest productivity under climate change? The uncertainty of CO_2_ fertilization and climate effects. Climate 8(12):141

[CR29] Tchebakova NM, Parfenova E, Korets M, Conard S (2016) Potential change in forest types and stand heights in central Siberia in a warming climate. Environ Res Lett 11:035016

[CR30] Ukrainetz NK, O’Neill GA, Jaquish B (2011) Comparison of fixed and focal point seed transfer systems for reforestation and assisted migration: a case study for interior spruce in British Columbia. Can J for Res 41:1452–1464

[CR31] Wang T, O’Neill GA, Aitken SN (2010) Integrating envrionmental and genetic effect to predict responses of tree population to climate. Ecol Appl 20:153–156 20349837 10.1890/08-2257.1

[CR32] Wang T, Hamann A, Spittlehouse DL, Murdock TQ (2012) ClimateWNA—high-resolution spatial climate data for Western North America. J Appl Meteorol Climatol 51:16–29

[CR33] Wang YH, Hogg EH, Price DT, Edwards J, Williamson T (2014) Past and projected future changes in moisture conditions in the Canadian boreal forest. For Chron 90:678–691

[CR34] Yang Y, Huang S, Vassov R, Pinno B, Chhin S (2019) Climate-sensitive height–age models for top height trees in natural and reclaimed oil sands stands in Alberta, Canada. Can J for Res. 10.1139/cjfr-2019-0293

[CR35] Ying CC, Yanchuk AD (2006) The development of British Columbia’s tree seed transfer guidelines: purpose, concept, methodology, and implementation. For Ecol Manage 227:1–13

[CR36] Ying CC, Illingworth K, Carlson M (1984) Geographic variation in lodgepole pine and its implications for tree improvement in British Columbia. Washington State University, Cooperative Extension Service, Pullman Washington, Spokane, Washington and Vancouver

[CR37] Zhao Y, Wang T (2023) Predicting the global fundamental climate niche of lodgepole pine for climate change adaptation. Front for Glob Change. 10.3389/ffgc.2023.1084797

